# Improvement and application of recommended food score for hypertension in Korean adults: the Korean Genome and Epidemiology Study

**DOI:** 10.3389/fnut.2024.1400458

**Published:** 2024-06-14

**Authors:** Jiyoung Hwang, Jeongsu Kim, Hyesook Kim

**Affiliations:** ^1^Department of Nutritional Science and Food Management, Graduate Program in System Health Science and Engineering, Ewha Womans University, Seoul, Republic of Korea; ^2^Department of Food and Nutrition, Wonkwang University, Iksan-si, Republic of Korea

**Keywords:** dietary approaches to stop hypertension diet, high blood pressure, hypertension, improved recommended food score for hypertension, Korean Genome and Epidemiology Study

## Abstract

**Background:**

Addressing dietary factors to lower blood pressure can be a crucial strategy at the population level to mitigate the risk of hypertension. In a prior investigation, a tailored food score was used as a dietary index relevant to hypertension among Korean adults. This current study aims to assess the association between the overall quality of the diet, taking into account more precise food components, and evaluate the risk of developing hypertension.

**Methods:**

This prospective cohort study included 5,342 adults aged 40–70 without hypertension who participated in the Korean Genome and Epidemiology Study (KoGES) from 2001 to 2016. The improved Recommended Food Score for Hypertension (iRFSH) is a modified version of the Recommended Food Score to assess the consumption of foods recommended in the Dietary Approaches to Stop Hypertension (DASH) diet for Korean foods. A higher score reflects greater consumption of recommended foods, indicative of higher dietary quality. The maximum total score is 65. High blood pressure, which includes both hypertension and prehypertension, was analyzed using Cox proportional hazard regression models to examine its prospective relationship with iRFSH.

**Results:**

Among 2,478 males and 2,864 females with 10.8 mean years of follow-up, a higher score of iRFSH was associated with a lower risk of hypertension in the highest quintile compared to the lowest quintile [total: hazard ratio (HR): 0.79; 95% confidence interval (CI): 0.72, 0.87; female: HR: 0.71; 95% CI: 0.62, 0.83].

**Conclusion:**

Higher iRFSH is associated with a lower incidence of hypertension. Our results suggest that the iRFSH may be a potential tool for assessing dietary quality and dietary patterns and predicting the risk of hypertension in Korean adults.

## 1 Introduction

Hypertension, or elevated blood pressure, is a medical condition associated with a significantly heightened risk of diseases of the heart, brain, kidney, and various other organs and a major contributor to premature death globally (1, 2). In 2021, hypertension was prevalent among 28.0% of Korean adults aged 20 and above, affecting around 12.3 million people (3). Of these, 5.3 million (43.5%) were 65 years old or older. The number of hypertension cases has risen significantly over the years, from 3.0 million in 2002 to 11.1 million in 2021 (3). A pivotal approach to preventing HBP involves scrutinizing and modifying dietary patterns to suit individual needs (4). It is rational to investigate the role of overall diet in disease risk, a methodology widely adopted in numerous studies aimed at comprehending the prevention and management of chronic diseases (5, 6). This effort necessitates integrating both the Dietary Approaches to Stop Hypertension (DASH) diet and a dietary quality index. While the DASH diet is specifically crafted to manage blood pressure, the dietary quality index serves the purpose of evaluating and promoting individual dietary habits for disease prevention (7, 8). DASH is a dietary pattern for HBP that has been emphasized in previous research (9).

Dietary patterns and quality can vary among populations due to factors such as food availability, socioeconomic status, residential area, ethnicity, and culture (10). According to existing literature, the traditional Korean diet is characterized by a predominant consumption of plant-derived products and a moderate intake of animal-derived food. It primarily includes grains (mainly rice), veges (mainly kimchi), seaweed, fish, and meat in moderation, resulting in a high-carbohydrate, low-fat diet (11, 12). Given the distinctions in dietary patterns between Koreans and individuals from other countries, the impact on health outcomes may differ.

The Recommended Food Score for Hypertension (RFSH) in the Korean adult population was developed by Han et al. based on the reported consumption of foods recommended in the DASH diet modified for Korean foods (13). It is a simple diet quality indicator for HBP with a score of 1 or 0, depending on the recommended food. However, this tool was developed without consideration of the nutrients in foods when creating the RFSH food list. Some of these nutrients, especially sodium, potassium, and saturated fat, are closely related to blood pressure. Therefore, it is necessary to change the food items in the sodium-rich food and red and processed meat categories because these foods are major sources of the main nutrients that cause hypertension.

**Figure 1:**
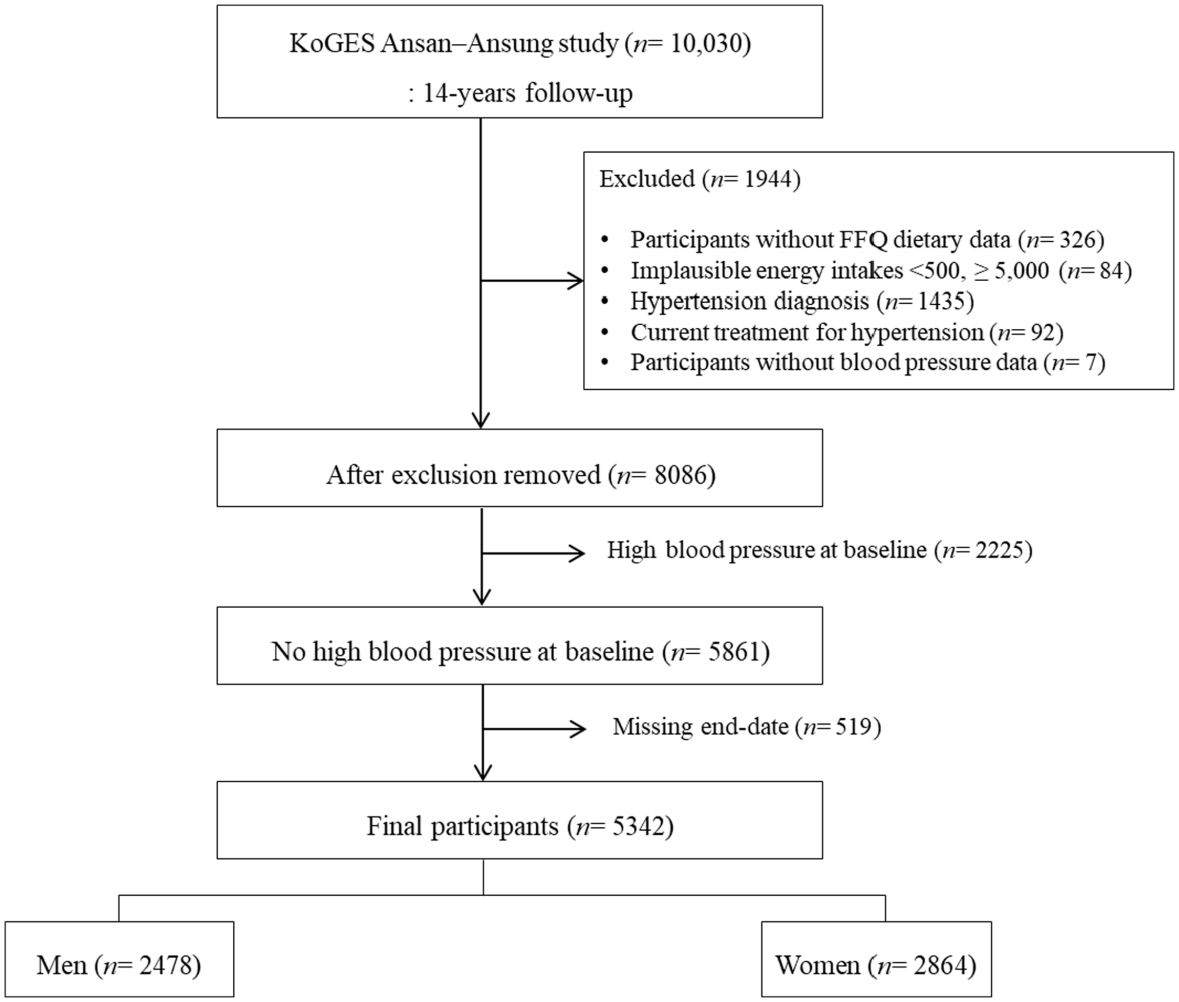
Study flow diagram. KoGES Korea Korean Genome and Epidemiology Study.

This study aimed to assess the relationship between overall dietary quality and hypertension risk in the Korean adult population. Using data from the Ansan–Ansung community-based cohort of the Korean Genome and Epidemiology Study (KoGES), we applied an improved RFSH (iRFSH) scoring system to prevent hypertension by examining its association with high blood pressure (see Figure 1).

## 2 Methods

### 2.1 Study design and population

The present study was conducted using data from the Ansan–Ansung cohort, an ongoing community-based prospective cohort. A comprehensive overview of the Ansan–Ansung cohort is available elsewhere (14). In summary, 10,030 participants aged 40–69 years who resided in the Ansan or Ansung regions of the Republic of Korea were recruited between 2001 and 2003 using a two-stage cluster sampling method. Detailed information was gathered at baseline and biennially through surveys, which involved structured interviews, health examinations, and laboratory tests.

Among the 10,030 participants, the following subjects were excluded from this study: those with missing dietary data (*n* = 326), with a daily calorie intake <500 kcal or ≥ 5,000 kcal (*n* = 84), diagnosed with hypertension (*n* = 1,435), having current treatment for hypertension (*n* = 92), and those without systolic blood pressure (SBP) and diastolic blood pressure (DBP) measurements (*n* = 7). Lastly, those with HBP at baseline (*n* = 2,225) and missing end-date (*n* = 519) were excluded. As a result, a final sample of 5,342 participants (2,478 males, 2,864 females) formed our study population.

The Ansan–Ansung study protocol was reviewed and approved by the Institutional Review Board of the Korea Centers for Disease Control and Prevention, and all study participants submitted written informed consent. The present study was approved by the Institutional Review Board of Ewha Womans University (approval no. E-202009-0008-01).

### 2.2 General characteristics

We obtained the demographic and socioeconomic characteristics of the participants, including age (years), sex (male/female), body mass index (BMI), education level, household income, alcohol consumption, smoking status, menopause status in females only (pre-menopause/post-menopause), physical activity, and blood pressure (SBP and DBP) values. BMI was calculated as weight (kg)/height (m^2^) and classified into one of the following four classes: underweight, normal, pre-obese, and obese. We then established four levels of education (elementary school or less/middle school/high school/college or above), two levels of household income (low/high), two levels of alcohol consumption (non-drinker/current drinker), and three levels of smoking status (non-smoker/former smoker/current smoker). Smoking was defined as having smoked more than 10 packs during the entire life. Daily total physical activity was calculated by summing the multiplication of each activity level by the time of physical activity and was scored as 1, 2, and 3 for light, moderate, and heavy activity, respectively. Energy intake was estimated (kcal/day).

### 2.3 Classification of HBP

The definition of hypertension is an SBP ≥ 140 mmHg, or DBP ≥ 90 mmHg, or self-reported use of antihypertensive medication (15). Prehypertension is defined as an SBP ≥ 120 and ≤ 140 mmHg and DBP ≥ 80 and ≤ 90 mmHg (16). Because this group of participants has a risk of developing heart disease, lifestyle measures to prevent the occurrence of HBP should be prepared. In this study, we defined HBP as including hypertension and prehypertension.

### 2.4 iRFSH

Dietary intake was investigated using a semi-quantitative food questionnaire (semi-FFQ). A total of 103 types of foods were displayed in the semi-FFQ and investigated with seven levels of frequency of intake (almost never/once a month/2–3 times a month/1–2 times a week/3–4 times a week/once a day/twice a day/three times a day) and three levels of average size for consumption (small/medium/large) for the previous year (17).

We reclassified three food groups (red and processed meats, low-sodium foods, and vegetables) based on the RFSH developed by Han et al. (13) to measure diet quality for hypertension. Among the food items contributing to the red and processed meat group, we excluded those with low saturated fat and cholesterol. We modified the sodium-rich food group to include only foods with a Na:K ratio ≤ 1.8 after selecting foods with a sodium intake ≥700 mg per serving based on nutritional data collected as part of the 112-item Food Frequency Questionnaire (FFQ) applied in the Korea National Health and Nutrition Examination Survey (KHNANES). Lastly, as kimchi is not only a source of sodium but also contains vitamin C, vitamin K, dietary fiber, and an abundance of lactic acid bacteria (18), it was removed from the sodium-rich food group and reclassified as a vegetable.

Participants’ dietary habits were evaluated using an iRFSH scoring system that assigned points based on their consumption of recommended and non-recommended foods. For recommended foods (whole grains, nuts & legumes, vegetables, fruits, milk, fish), participants received 1 point for each food group they consumed more than once a week. Conversely, for non-recommended foods (processed meats, sugary drinks, salty foods), participants received 0 points if they consumed a non-recommended food group more than once a week but got 1 point if they consumed it less often. The total iRFSH score, ranging from 0 to 65, reflected participants’ overall adherence to a healthy diet, with higher scores indicating a higher consumption of recommended foods and lower consumption of non-recommended foods. Table 1 provides a comprehensive list of all foods or food groups within these nine categories.

**Table 1:** Food groups and foods included in iRFSH for KoGES Ansan–Ansung study.^a^

Food groups	Number of foods included	Foods included
Whole grains	1	Multigrain rice
Nuts and legumes	4	Soybeans, peanut/almond/pine nut, soy milk, tofu (including soft tofu, tofu stew, and soybean paste stew)
Fruits/Fruit juices	11	Strawberry, oriental melon/melon, watermelon, peach/plum, banana, persimmon/dried persimmon, mandarin, pear/pear juice, apple/apple juice, orange/orange juice, grape/grape juice
Vegetables	25	Green-bean sprouts/bean sprouts, spinach, balloon flower/burdock, green young pumpkin, pumpkin/pumpkin porridge, cucumber, daikon (soup, braised)/pickled radish, other green vegetables (shepherd’s purse, mugwort, mustard, curled mallow, *chwinamul*, the green part of a radish), bracken/sweet potato stem/taro stem, cabbage/cabbage soup, oyster mushroom, other mushrooms (button, enoki, shiitake), tomato/tomato juice, lettuce, perilla leaf, chives/water parsley, green chili, pepper leaves, onion, vegetable juice/green juice, carrot/carrot juice, cabbage kimchi, *kkakdugi*/radish kimchi, *dongchimi/*radish water kimchi, other kimchi (green onion kimchi, mustard pickles, Korean lettuce kimchi)
Milk	1	Milk
Fish	7	Blueback fish (mackerel, saury, Japanese Spanish mackerel), hairtail, stir-fried anchovy/anchovies, croaker, pollack/frozen pollack/dried pollack, eel, raw fish (sand dab, flatfish, tuna, rockfish, etc.)
Red and processed meats	7	Pork/grilled pork belly, boiled pork (*jangjorim*, *jokbal*, Korean sausage), roast pork (galbi, sirloin), roast beef, ham/sausage, intestine meat, braised ribs
Sugar-sweetened beverages	3	Carbonated beverage (cola, sprite, carbonated fruit beverage), other drinks (*sikhye*, citron tea, etc.), coffee with sugar
Sodium-rich foods	6	*Naengmyeon*/buckwheat noodles, noodles/chopped noodles/*udon*, black bean paste noodles, white rice cake/rice cake soup, dumplings, Korean instant noodles (*ramyeon*)

a iRFSH, improved Recommended Food Score for Hypertension; KoGES, Korean Genome and Epidemiology Study.

### 2.5 Statistical analysis

The participants were categorized into quintiles according to their iRFSH by gender. To understand their general characteristics, Student *t*-tests and χ^2^-tests were conducted, taking the types of variables into account. To examine the associations between the quintiles of iRFSH and hypertension incidence, we applied Cox proportional hazard regression and presented them as hazard ratios (HRs) and 95% confidence intervals (CIs). The person-time of follow-up was calculated from the date of the baseline examination until the date of incident hypertension, censoring, or the date of the last examination, whichever came first. The average follow-up time was 75.1 months, resulting in 33,521 person-years. A multivariable model was adjusted for age, sex, BMI, education level, household income, drinking status, smoking status, regular exercise, energy intake (kcal/day), and menopause status (females only). To test for potential linear trends, we treated each iRFSH quintile as a continuous variable. Two-sided *p*-values less than 0.05 were accepted as statistically significant. All statistical analyses were performed using the SAS package version 9.4 (SAS Institute, Inc., Cary, NC, United States).

## 3 Results

### 3.1 Characteristics of the study population

Table 2 and Supplementary Table S1 characterize the participants by the iRFSH quintiles. In total, 5,342 participants were recruited in this study, with a mean age of 50.27 years in males and 50.37 years in females. Females showed a slightly higher iRFSH score compared to males. In the higher iRFSH quintile groups, females tended to be younger (*p*-trend <0.0001) than those in the lower iRFSH quintile. Moving from Q1 (least healthy) to Q5 (healthiest), both male and female participants showed a tendency toward higher education and household income levels, along with increased energy intake. Male participants were less likely to be current smokers (*p*-trend <0.05), and female participants were less likely to be post-menopausal (*p*-trend <0.05).

**Table 2:** Sociodemographic and lifestyle characteristics across quintiles of iRFSH in the KoGES Ansan–Ansung study.^a^

	Total (*n* = 5,342)			Male (*n* = 2,478)			Female (*n* = 2,864)		
	Quintile 1	Quintile 3	Quintile 5	Quintile 1	Quintile 3	Quintile 5	Quintile 1	Quintile 3	Quintile 5
*n*	1,108	871	1,118	615	412	432	493	459	686
Score range	13–23	28–30	36–59	13–23	28–30	36–53	15–23	28–30	36–59
Age (years)	50.95 ± 8.88	50.32 ± 8.26	49.68 ± 8.11**	50.62 ± 8.74	50.27 ± 8.29	50.25 ± 8.29	51.35 ± 9.06	50.37 ± 8.24	49.32 ± 7.98**
BMI (kg/m^2^)	23.90 ± 2.93	24.12 ± 2.89	24.29 ± 2.89*	23.82 ± 2.79	23.91 ± 2.87	24.07 ± 2.73	24.00 ± 3.10	24.29 ± 2.90	24.43 ± 2.98
**Education level, *n*(%)**									
Elementary school or less	347 (31.32)	223 (25.60)	240 (21.47)**	121 (19.67)	72 (17.48)	51 (11.81)*	226 (45.84)	151 (32.90)	189 (27.55)**
Middle school	238 (21.48)	216 (24.80)	262 (23.43)	127 (20.65)	85 (20.63)	84 (19.44)	111 (22.52)	131 (28.54)	178 (25.95)
High school	352 (31.77)	293 (33.64)	432 (38.64)	228 (37.07)	156 (37.86)	185 (42.82)	124 (25.15)	137 (29.85)	247 (36.01)
College or above	162 (14.62)	136 (15.61)	181 (16.19)	137 (22.28)	98 (23.79)	112 (25.93)	25 (5.07)	38 (8.28)	69 (10.06)
No response	9 (0.81)	3 (0.34)	3 (0.27)	2 (0.33)	1 (0.24)	0 (0.00)	7 (1.42)	2 (0.44)	3 (0.44)
Household income level, *n*(%)									
Lower	381 (34.39)	243 (27.90)	249 (22.27)**	167 (27.15)	95 (23.06)	79 (18.29)**	214 (43.41)	148 (32.24)	170 (24.78)**
Lower middle	339 (30.60)	250 (28.70)	343 (30.68)	203 (33.01)	120 (29.13)	133 (30.79)	136 (27.59)	130 (28.32)	210 (30.61)
Upper middle	315 (28.43)	282 (32.38)	393 (35.15)	203 (33.01)	150 (36.41)	159 (36.81)	112 (22.72)	132 (28.76)	234 (34.11)
High	57 (5.14)	81 (9.30)	120 (10.73)	39 (6.34)	45 (10.92)	60 (13.89)	18 (3.65)	36 (7.84)	60 (8.75)
No response	16 (1.44)	15 (1.72)	13 (1.16)	3 (0.49)	2 (0.49)	1 (0.23)	13 (2.64)	13 (2.83)	12 (1.75)
**Drinking status, *n*(%)**									
Non-drinker	461 (41.61)	396 (45.46)	568 (50.81)**	128 (20.81)	80 (19.42)	95 (21.99)	333 (67.55)	316 (68.85)	473 (68.95)
Former drinker	80 (7.22)	51 (5.86)	56 (5.01)	65 (10.57)	43 (10.44)	39 (9.03)	15 (3.04)	8 (1.74)	17 (2.48)
Current drinker	564 (50.90)	424 (48.68)	490 (43.83)	422 (68.62)	289 (70.15)	297 (68.75)	142 (28.80)	135 (29.41)	193 (28.13)
No response	3 (0.27)	0 (0.00)	4 (0.36)	0 (0.00)	0 (0.00)	1 (0.23)	3 (0.61)	0 (0.00)	3 (0.44)
**Smoking status, *n*(%)**									
Non-smoker	564 (50.90)	502 (57.63)	753 (67.35)**	104 (16.91)	69 (16.75)	106 (24.54)*	460 (93.31)	433 (94.34)	647 (94.31)
Former smoker	190 (17.15)	133 (15.27)	138 (12.34)	183 (29.76)	127 (30.83)	134 (31.02)	7 (1.42)	6 (1.31)	4 (0.58)
Current smoker	347 (31.32)	230 (26.41)	216 (19.32)	328 (53.33)	216 (52.43)	191 (44.21)	19 (3.85)	14 (3.05)	25 (3.64)
No response	7 (0.63)	6 (0.69)	11 (0.98)	0 (0.00)	0 (0.00)	1 (0.23)	7 (1.42)	6 (1.31)	10 (1.46)
**Physical activity, *n*(%)**									
None	902 (81.41)	716 (82.20)	908 (81.22)	492 (80.00)	327 (79.37)	338 (78.24)	410 (83.16)	389 (84.75)	570 (83.09)
Light (<1 h/day)	170 (15.34)	127 (14.58)	160 (14.31)	104 (16.91)	69 (16.75)	68 (15.74)	66 (13.39)	58 (12.64)	92 (13.41)
Moderate (<2 h/day)	19 (1.71)	20 (2.30)	30 (2.68)	10 (1.63)	12 (2.91)	13 (3.01)	9 (1.83)	8 (1.74)	17 (2.48)
Heavy (≥2 h/day)	11 (0.99)	4 (0.46)	15 (1.34)	7 (1.14)	2 (0.49)	10 (2.31)	4 (0.81)	2 (0.44)	5 (0.73)
No response	6 (0.54)	4 (0.46)	5 (0.45)	2 (0.33)	2 (0.49)	3 (0.69)	4 (0.81)	2 (0.44)	2 (0.29)
**Menopause status, *n*(*%*)**									
Pre-menopause	─	─	─	─	─	─	259 (52.54)	257 (55.99)	414 (60.35)*
Post-menopause	─	─	─	─	─	─	234 (47.46)	202 (44.01)	272 (39.65)
Energy intake (kcal/day)	1651.06 ± 479.48	1898.21 ± 521.33	2326.60 ± 665.83**	1746.41 ± 479.50	1975.28 ± 516.20	2407.19 ± 591.36**	1532.10 ± 452.54	1829.03 ± 516.74	2275.85 ± 704.43**

a Values are means ± SD or *n*(%). Only results for quintiles 1, 3, and 5 are shown; see Supplementary Table S1 for results across all quintiles. *p*-trends were assessed by modeling the median value of the quintiles (continuous) or with the use of the Mantel–Haenszel χ^2^-test for linear trends (categorical). *p-trend < 0.05; **p-trend < 0.0001. iRFSH, improved Recommended Food Score for Hypertension; KoGES, Korean Genome and Epidemiology Study; SD, standard deviation.

### 3.2 The mean score of food groups according to quintiles of iRFSH

The scores (mean ± SD) of each food group included in the iRFSH of total participants, males, and females, respectively, are shown in Table 3 and Supplementary Table S2. The mean scores for each recommended food group in the iRFSH, which includes whole grains, nuts and legumes, vegetables, fruits/fruit juices, milk, and fish, showed an increasing trend across higher quintiles of iRFSH for both males and females (*p*-trend <0.0001). Conversely, the mean scores of each unrecommended food group in iRFSH, such as sugar-sweetened beverages, red and processed meats, and sodium-rich foods, tended to decrease across the higher quintiles of iRFSH both in males and females (*p*-trend <0.0001 for both genders; *p*-trend <0.0001 for sugar-sweetened beverages and red and processed meats, and *p*-trend <0.05 for sodium-rich foods).

**Table 3:** Mean score of each recommended food group in iRFSH in the KoGES Ansan–Ansung study.^a^

	Total (*n* = 5,342)			Male (*n* = 2,478)			Female (*n* = 2,864)		
	Quintile 1	Quintile 3	Quintile 5	Quintile 1	Quintile 3	Quintile 5	Quintile 1	Quintile 3	Quintile 5
Score range	13–23	28–30	36–59	13–23	28–30	36–53	15–23	28–30	36–59
Score out of 65	20.55 ± 2.12	28.94 ± 0.82	40.10 ± 3.71**	20.40 ± 2.22	28.95 ± 0.82	39.78 ± 3.42**	20.75 ± 1.98	28.93 ± 0.83	40.30 ± 3.87**
Whole grains, score out of 1	0.40 ± 0.49	0.57 ± 0.49	0.69 ± 0.46**	0.34 ± 0.48	0.52 ± 0.50	0.63 ± 0.48**	0.46 ± 0.50	0.62 ± 0.49	0.73 ± 0.45**
Nuts and legumes, score out of 4	0.81 ± 0.77	1.44 ± 0.83	1.94 ± 0.89**	0.90 ± 0.79	1.49 ± 0.87	2.08 ± 0.91**	0.70 ± 0.72	1.39 ± 0.79	1.86 ± 0.87**
Vegetables, score out of 25	4.47 ± 2.22	9.70 ± 2.60	15.74 ± 3.44**	4.75 ± 2.35	10.35 ± 2.55	16.48 ± 3.47**	4.11 ± 1.98	9.10 ± 2.49	15.28 ± 3.35**
Fruits/Fruit juices, score out of 11	0.64 ± 1.13	2.21 ± 2.16	5.77 ± 3.14**	0.60 ± 1.08	1.95 ± 2.10	5.16 ± 3.30**	0.69 ± 1.18	2.45 ± 2.18	6.16 ± 2.97**
Milk, score out of 1	0.33 ± 0.47	0.53 ± 0.50	0.67 ± 0.47**	0.33 ± 0.47	0.49 ± 0.50	0.66 ± 0.47**	0.33 ± 0.47	0.56 ± 0.50	0.68 ± 0.47**
Fish, score out of 7	0.52 ± 0.89	1.53 ± 1.29	2.86 ± 1.67**	0.60 ± 0.97	1.64 ± 1.39	3.08 ± 1.81**	0.43 ± 0.76	1.44 ± 1.19	2.72 ± 1.56**
Sugar-sweetened beverages, score out of 3	1.65 ± 0.57	1.58 ± 0.62	1.45 ± 0.70**	1.53 ± 0.63	1.45 ± 0.68	1.29 ± 0.76**	1.81 ± 0.44	1.70 ± 0.53	1.56 ± 0.63**
Red and processed meats, score out of 7	6.30 ± 1.17	6.00 ± 1.29	5.61 ± 1.52**	6.13 ± 1.26	5.87 ± 1.39	5.32 ± 1.64**	6.52 ± 1.00	6.12 ± 1.19	5.79 ± 1.41**
Sodium-rich foods, score out of 6	5.18 ± 1.03	5.10 ± 1.06	5.07 ± 1.14*	5.02 ± 1.08	4.95 ± 1.13	4.85 ± 1.23*	5.38 ± 0.92	5.23 ± 0.97	5.21 ± 1.06*

a Values are means ± SD or *n*(%). Only results for quintiles 1, 3, and 5 are shown; see Supplementary Table S2 for results across all quintiles. *p*-trends were assessed by modeling the median value of the quintiles. *p-trend < 0.05; **p-trend < 0.0001. iRFSH, improved Recommended Food Score for Hypertension; KoGES, Korean Genome and Epidemiology Study; SD, standard deviation.

### 3.3 Means and standard deviations of SBP and DBP according to quintiles of iRFSH

Table 4 and Supplementary Table S3 show the mean and standard deviation (SD) of SBP and DBP in each quintile of the iRFSH after adjusting for potential covariates. Model 1 included adjustments for sex, age, and menopause status (females only), and Model 2 was further adjusted for BMI, education level, household income, drinking status, smoking status, and regular exercise. In Model 1, the mean SBP tended to decrease across higher quintiles of iRFSH in both males and females (*p*-trend <0.05). Similarly, in Model 2, a decreasing trend was observed in both genders (*p*-trend <0.05). Furthermore, in Model 1, participants in the higher quintiles of iRFSH had a lower mean DBP compared to those in the lower quintiles of iRFSH in both males and females (*p*-trend <0.05). In Model 2, the mean DBP tended to decrease across higher quintiles of iRFSH in both genders (*p*-trend <0.05).

**Table 4:** Mean values and standard deviations of SBP and DBP across quintiles of iRFSH in the KoGES Ansan–Ansung study.^a^

	Total (*n* = 5,342)			Male (*n* = 2,478)			Female (*n* = 2,864)		
	Quintile 1	Quintile 3	Quintile 5	Quintile 1	Quintile 3	Quintile 5	Quintile 1	Quintile 3	Quintile 5
**SBP**									
Model 1^b^	113.99 ± 0.62	112.47 ± 0.68	111.43 ± 0.63**	114.69 ± 0.82	113.62 ± 0.75	113.18 ± 0.66*	113.12 ± 0.69	112.44 ± 0.63	111.35 ± 0.61**
Model 2^c^	114.49 ± 0.64	113.03 ± 0.66	111.99 ± 0.61**	115.14 ± 0.80	114.07 ± 0.75	113.01 ± 0.68*	113.67 ± 0.67	113.10 ± 0.62	111.92 ± 0.63**
**DBP**									
Model 1^b^	75.37 ± 0.64	74.89 ± 0.62	73.31 ± 0.67**	77.44 ± 0.67	76.23 ± 0.64	76.21 ± 0.70**	74.06 ± 0.66	73.7 ± 0.60	73.13 ± 0.61*
Model 2^c^	75.71 ± 0.63	75.22 ± 0.57	73.74 ± 0.68**	77.73 ± 0.67	76.58 ± 0.67	75.58 ± 0.69**	74.44 ± 0.67	73.14 ± 0.61	72.57 ± 0.66**

a Values are presented as means ± SD. Only results for quintiles 1, 3, and 5 are shown; see Supplementary Table S3 for results across all quintiles. *p*-trends were assessed using general linear models for continuous variables after adjustment.

b Model 1: adjusted by sex, age, and menopause status (females only).

c Model 2: adjusted by age, sex, BMI (underweight/normal/pre-obese/obese class), education level (elementary school or less/middle school/high school/college or above), household income (low/high), drinking status (non-drinker/current drinker), smoking status (non-smoker/former smoker/current smoker), regular exercise (light activities/moderate-intensity activities/heavy-intensity activities), energy intake (kcal/day), and menopause status [pre-menopause/post-menopause (females only)].

*p-trend < 0.05; **p-trend < 0.0001. iRFSH, improved Recommended Food Score for Hypertension; KoGES, Korean Genome and Epidemiology Study; SD, standard deviation; SBP, systolic blood pressure; DBP, diastolic blood pressure.

### 3.4 Relationship between iRFSH and risk of incidence of HBP

The HRs and 95% CIs for incidence of HBP across quintiles of iRFSH are presented in Table 5 and Supplementary Table S4. When the iRFSH was analyzed as a continuous variable, a significant inverse relationship with HBP was observed, except in males. In Model 2, a 1-point increase in iRFSH was associated with a 6.0% reduction in the prevalence of HBP in the total participants (HR: 0.94, 95% CI: 0.90–0.97) and a 4.0% reduction in females (HR: 0.96, 95% CI: 0.86–0.99), indicating a decrease in risk as iRFSH scores increase. In the total participants, HRs for HBP across quintiles in Model 1 and Model 2 were 0.83 (95% CI, 0.74–0.93, *p*-trend <0.05) and 0.79 (95% CI, 0.72–0.87, *p*-trend <0.001), respectively. Among females, the risk of HBP in Model 1 and Model 2 decreased with HRs of 0.73 (95% CI, 0.61–0.88, *p*-trend <0.05) and 0.71 (95% CI, 0.62–0.83, *p*-trend <0.001), respectively. However, in males, there was no significant association between iRFSH and the risk of HBP incidence.

**Table 5:** HRs and 95% CIs for hypertension according to the quintiles of iRFSH in the KoGES Ansan–Ansung study^a^.

	Total (*n* = 5,342)				Male (*n* = 2,478)				Female (*n* = 2,864)			
	Continuous	Quintile 1	Quintile 3	Quintile 5	Continuous	Quintile 1	Quintile 3	Quintile 5	Continuous	Quintile 1	Quintile 3	Quintile 5
Person-years, follow-up	57896.6	11958.3	9548.3	12053.4	26078.2	6477.1	4285.5	4579.4	31818.4	5481.2	5262.8	7,474
Model 1^b^	0.98 (0.87, 1.03)	1 (reference)	0.98 (0.87, 1.09)	0.83 (0.74, 0.93)*	1.03 (0.98, 1.08)	1 (reference)	0.96 (0.83, 1.13)	0.95 (0.82, 1.10)	0.98 (0.91, 0.99)	1 (reference)	1.00 (0.83, 1.19)	0.73 (0.61, 0.88)*
Model 2^c^	0.94 (0.90, 0.97)	1 (reference)	1.00 (0.89, 1.12)	0.79 (0.72, 0.87)**	0.99 (0.89, 1.07)	1 (reference)	0.96 (0.82, 1.13)	0.95 (0.80, 1.11)	0.96 (0.86, 0.99)	1 (reference)	1.04 (0.87, 1.26)	0.71 (0.62, 0.83)**

a Values are presented as HRs and 95% CIs. Only results for quintiles 1, 3, and 5 are shown; see Supplementary Table S4 for results across all quintiles. For calculating *p*-trends, the RFSH was used in its continuous form.

b Model 1: adjusted by age, sex, and menopause status [pre-menopause/post-menopause (females only)].

c Model 2: adjusted by age, sex, BMI (underweight/normal/pre-obese/obese class), education level (elementary school or less/middle school/high school/college or above), household income (low/high), drinking status (non-drinker/current drinker), smoking status (non-smoker/former smoker/current smoker), regular exercise (light activities/moderate-intensity activities/heavy-intensity activities), energy intake (kcal/day), and menopause status [pre-menopause/post-menopause (females only)].

*p-trend < 0.05; **p-trend < 0.0001. iRFSH, improved Recommended Food Score for Hypertension; KoGES, Korean Genome and Epidemiology Study; HR, hazard ratio; CI, confidence interval.

## 4 Discussion

In this study, we improved the RFSH food list for hypertension in Korean adults and reaffirmed the association between HBP and the iRFSH score using data from a prospective cohort study conducted on a large population sample from Korea. The association between the RFSH dietary pattern and hypertension has been examined previously by Han et al. (13). Our study used data on RFSH food consumption from 2001 baseline to 2014 follow-up, providing a comprehensive analysis of the prospective link between the modified RFSH diet score and hypertension risk in Korean adults. Strengths of our study include its extended follow-up duration, allowing for the identification of long-term HBP risks, and its use of data from large community-based cohorts.

The modification of the iRFSH food list involved three key adjustments. First, within the red and processed meat group, four foods (stock soup of bone and stew meat/beef-bone soup, pork back-bone stew, beef stew/hot spicy meat stew/radish soup) were excluded. Stock soups or stews consist primarily of broth or a combination of meat and vegetables, so they may not typically be considered processed meats (19). Second, the sodium-rich food group was refined. The original RFSH reported the frequency of sodium-rich food intake but lacked quantitative sodium content. The modification involved selecting foods with a Na:K ratio ≥ 1.8, considering the established association between a lower Na:K ratio and better blood pressure control in Korean adults (20, 21). Lastly, cabbage kimchi and other types of kimchi, initially categorized as sodium-rich foods, were re-evaluated. With a sodium content of approximately 200 mg and recognizing the diverse health benefits of kimchi, including weight reduction (22), improved body composition (23), and cardiovascular health (18), these items were deemed more fitting for inclusion in the vegetable group rather than the sodium-rich food category (22). This decision was based on the nutrient composition of kimchi (24), which includes dietary fiber, vitamins, calcium, capsaicin, and niacin, contributing to its potential positive effects on blood pressure and overall health.

In this study, participants with higher iRFSH scores consumed recommended foods more frequently. These foods include whole grains, nuts, legumes, vegetables, fruits/fruit juices, milk, and fish. Conversely, they consumed less of the unrecommended foods, such as sugar-sweetened beverages, red and processed meats, and sodium-rich foods, compared to those with lower iRFSH scores. The food groups consistent with the iRFSH closely resemble the DASH diet. These dietary patterns emphasize the intake of fruits, vegetables, seafood, legumes, nuts, and low-fat dairy products while restricting the consumption of red and processed meats, saturated fatty acids, sodium, and added sugars (9, 25). Recent studies indicate that adhering to these healthy dietary patterns is linked to a significant reduction in the risk of hypertension (13, 26–29). Furthermore, the findings of this study lend support to the positive effects of the traditional Korean food, characterized by substantial kimchi consumption. Kimchi is globally appraised as a healthy food with antioxidant and anti-inflammatory activities (30). Additionally, processed meats typically contain high levels of saturated and trans fats, contributing to cardiovascular disease risk (31). Therefore, reducing processed meat consumption, as advocated by the iRFSH approach, may be linked to lower hypertension risk.

Dietary food scores are proxy indicators of actual food consumption and daily eating habits, offering an alternative approach to comprehending the relationship between diet and disease risk (32). Our study findings showed that the mean SBP and DBP among subjects decreased significantly as their dietary pattern scores increased from Q1 to Q5 of the iRFSH. This trend aligns with the observed impact of the DASH diet, which demonstrated the greatest overall reduction in SBP and DBP (33). Notably, our findings indicate a mean blood pressure difference of approximately 2.5 mmHg between individuals with lower and higher quintiles of iRFSH. This difference may seem small, but it is clinically relevant because even a 2 mmHg reduction in SBP can decrease mortality from heart diseases and vascular diseases in middle-aged people by 7% (34).

The HRs were found to be significant in the total population as a result of this study. However, when analyzed by gender, the HRs were significant only in females in the correlation between iRFSH and hypertension. There is evidence for a gender difference in the association between eating habits and disease (35, 36). Many biological, pathological, and social determinants can contribute to the difference (37). For example, sex hormones, such as estrogen, may increase the release of nitrogen oxide and affect vascular smooth muscle contraction or relaxation (38). Estrogen can also affect the renin-angiotensin system, which can be regulated differently in males and females, and endogenous estrogen inhibits the expression of angiotensin receptor type 1 and angiotensinogen synthesis (39). Therefore, gender differences should be considered in analyzing the association between hypertension and dietary quality scores.

This study has several limitations. According to Plummer and Kaaks (40), the findings may not be generalizable to other societies, given the use of an FFQ based on local food supplies and a national survey. Hence, these results are primarily applicable to Korean adults. Moreover, the iRFSH is simply an indicator of diet quality related to hypertension and does not reflect total energy intake or energy requirements. Nevertheless, the simplicity of the score calculation based on DASH diet recommendations, coupled with validation in a cohort study, makes it valuable for providing information to individuals at risk of hypertension. Lastly, in this prospective study, the follow-up intervals were not uniform across all participants. The range for follow-up durations varied from 1.4 to 14.7 years (median, 13.5 years; mean, 10.8 years). This variation implies that the effect of independent variables may not be consistent across all participants. Despite these limitations, this prospective cohort study showed that measuring diet quality by the iRFSH index developed in this study may be useful in future research on dietary patterns and interventions as the primary prevention approach for reducing the risk of incident hypertension.

## 5 Conclusion

In conclusion, this is the first study to investigate the association between overall diet quality and the follow-up incidence of HBP in Korean adults. Understanding how the iRFSH score can be associated with hypertension will help develop dietary pattern strategies to prevent hypertension and related chronic diseases. Further studies with relatively larger sample sizes are needed to improve knowledge about the association between the iRFSH and HBP in the future.

## Data availability statement

The raw data supporting the conclusions of this article will be made available by the authors, without undue reservation.

## Ethics statement

The studies involving humans were approved by the present study was exempt from review by the Institutional Review Board (IRB) of Ewha Womans University (IRB No. E-202009-0008-01). All subjects signed informed consent forms. This retrospective study relied on the data from the KoGES from 2001 to 2016. The studies were conducted in accordance with the local legislation and institutional requirements. The participants provided their written informed consent to participate in this study.

## Author contributions

JH: Data curation, Investigation, Validation, Visualization, Writing – original draft, Writing – review & editing. JK: Data curation, Formal analysis, Writing – original draft. HK: Conceptualization, Data curation, Formal analysis, Funding acquisition, Investigation, Methodology, Project administration, Writing – original draft, Writing – review & editing.
