# Supplementary material for: Improvement and application of recommended food score for hypertension in Korean adults: the Korean Genome and Epidemiology Study
Source: Front Nutr. 2024 Jun 14;11:1400458. doi: 10.3389/fnut.2024.1400458 (PMC11211396; doi:10.3389/fnut.2024.1400458)
Supplement: Supplementary file 1 [file Table_1.DOCX]

| **Supplemental Table 1** Sociodemographic and lifestyle characteristics across quintiles of iRFSH of the KoGES Ansan–Ansung study**^1^** | | | | | | | | | | | | | | | | | |
| --- | --- | --- | --- | --- | --- | --- | --- | --- | --- | --- | --- | --- | --- | --- | --- | --- | --- |
|  | Total (*n*= 5,342) | | | | | | Male (*n*= 2,478) | | | | | | Female (*n*= 2,864) | | | | |
|  | Quintile 1 | Quintile 2 | Quintile 3 | Quintile 4 | Quintile 5 | Quintile 1 | | Quintile 2 | Quintile 3 | Quintile 4 | Quintile 5 | Quintile 1 | | Quintile 2 | Quintile 3 | Quintile 4 | Quintile 5 |
| *n* | 1108 | 1135 | 871 | 1110 | 1118 | 615 | | 556 | 412 | 463 | 432 | 493 | | 579 | 459 | 647 | 686 |
| Score range | 13 – 23 | 24 – 27 | 28 – 30 | 31 – 35 | 36 – 59 | 13 – 23 | | 24 – 27 | 28 – 30 | 31 – 35 | 36 – 53 | 15 – 23 | | 24 – 27 | 28 – 30 | 31 – 35 | 36 – 59 |
| Age (years) | 50.95 ± 8.88 | 50.75 ± 8.61 | 50.32 ± 8.26 | 49.76 ± 8.18 | 49.68 ± 8.11** | 50.62 ± 8.74 | | 50.48 ± 8.46 | 50.27 ± 8.29 | 50.45 ± 8.38 | 50.25 ± 8.29 | 51.35 ± 9.06 | | 51.02 ± 8.74 | 50.37 ± 8.24 | 49.27 ± 8.00 | 49.32 ± 7.98** |
| BMI (kg/m^2^) | 23.90 ± 2.93 | 24.10 ± 3.03 | 24.12 ± 2.89 | 24.15 ± 2.86 | 24.29 ± 2.89* | 23.82 ± 2.79 | | 23.82 ± 2.87 | 23.91 ± 2.87 | 23.99 ± 2.63 | 24.07 ± 2.73 | 24.00 ± 3.10 | | 24.37 ± 3.15 | 24.29 ± 2.90 | 24.27 ± 3.01 | 24.43 ± 2.98 |
| Education level, *n*(%) |  |  |  |  |  |  | |  |  |  |  |  | |  |  |  |  |
| Elementary school or less | 347 (31.32) | 362 (31.89) | 223 (25.60) | 260 (23.42) | 240 (21.47)** | 121 (19.67) | | 105 (18.88) | 72 (17.48) | 71 (15.33) | 51 (11.81)* | 226 (45.84) | | 257 (44.39) | 151 (32.90) | 189 (29.21) | 189 (27.55)** |
| Middle school | 238 (21.48) | 239 (21.06) | 216 (24.80) | 290 (26.13) | 262 (23.43) | 127 (20.65) | | 120 (21.58) | 85 (20.63) | 113 (24.41) | 84 (19.44) | 111 (22.52) | | 119 (20.55) | 131 (28.54) | 177 (27.36) | 178 (25.95) |
| High school | 352 (31.77) | 372 (32.78) | 293 (33.64) | 397 (35.77) | 432 (38.64) | 228 (37.07) | | 206 (37.05) | 156 (37.86) | 172 (37.15) | 185 (42.82) | 124 (25.15) | | 166 (28.67) | 137 (29.85) | 225 (34.78) | 247 (36.01) |
| College or above | 162 (14.62) | 159 (14.01) | 136 (15.61) | 159 (14.32) | 181 (16.19) | 137 (22.28) | | 123 (22.12) | 98 (23.79) | 104 (22.46) | 112 (25.93) | 25 (5.07) | | 36 (6.22) | 38 (8.28) | 55 (8.50) | 69 (10.06) |
| No response | 9 (0.81) | 3 (0.26) | 3 (0.34) | 4 (0.36) | 3 (0.27) | 2 (0.33) | | 2 (0.36) | 1 (0.24) | 3 (0.65) | 0 (0.00) | 7 (1.42) | | 1 (0.17) | 2 (0.44) | 1 (0.15) | 3 (0.44) |
| Household income level, *n*(%) |  |  |  |  |  |  | |  |  |  |  |  | |  |  |  |  |
| Lower | 381 (34.39) | 391 (34.45) | 243 (27.90) | 278 (25.05) | 249 (22.27)** | 167 (27.15) | | 154 (27.70) | 95 (23.06) | 104 (22.46) | 79 (18.29)** | 214 (43.41) | | 237 (40.93) | 148 (32.24) | 174 (26.89) | 170 (24.78)** |
| Lower middle | 339 (30.60) | 326 (28.72) | 250 (28.70) | 333 (30.00) | 343 (30.68) | 203 (33.01) | | 164 (29.50) | 120 (29.13) | 145 (31.32) | 133 (30.79) | 136 (27.59) | | 162 (27.98) | 130 (28.32) | 188 (29.06) | 210 (30.61) |
| Upper middle | 315 (28.43) | 321 (28.28) | 282 (32.38) | 369 (33.24) | 393 (35.15) | 203 (33.01) | | 184 (33.09) | 150 (36.41) | 150 (32.40) | 159 (36.81) | 112 (22.72) | | 137 (23.66) | 132 (28.76) | 219 (33.85) | 234 (34.11) |
| High | 57 (5.14) | 82 (7.22) | 81 (9.30) | 116 (10.45) | 120 (10.73) | 39 (6.34) | | 46 (8.27) | 45 (10.92) | 62 (13.39) | 60 (13.89) | 18 (3.65) | | 36 (6.22) | 36 (7.84) | 54 (8.35) | 60 (8.75) |
| No response | 16 (1.44) | 15 (1.32) | 15 (1.72) | 14 (1.26) | 13 (1.16) | 3 (0.49) | | 8 (1.44) | 2 (0.49) | 2 (0.43) | 1 (0.23) | 13 (2.64) | | 7 (1.21) | 13 (2.83) | 12 (1.85) | 12 (1.75) |
| Drinking status, *n*(%) |  |  |  |  |  |  | |  |  |  |  |  | |  |  |  |  |
| Non-drinker | 461 (41.61) | 497 (43.79) | 396 (45.46) | 531 (47.84) | 568 (50.81)** | 128 (20.81) | | 100 (17.99) | 80 (19.42) | 95 (20.52) | 95 (21.99) | 333 (67.55) | | 397 (68.57) | 316 (68.85) | 436 (67.39) | 473 (68.95) |
| Former-drinker | 80 (7.22) | 72 (6.34) | 51 (5.86) | 64 (5.77) | 56 (5.01) | 65 (10.57) | | 54 (9.71) | 43 (10.44) | 45 (9.72) | 39 (9.03) | 15 (3.04) | | 18 (3.11) | 8 (1.74) | 19 (2.94) | 17 (2.48) |
| Current-drinker | 564 (50.90) | 559 (49.25) | 424 (48.68) | 512 (46.13) | 490 (43.83) | 422 (68.62) | | 400 (71.94) | 289 (70.15) | 323 (69.76) | 297 (68.75) | 142 (28.80) | | 159 (27.46) | 135 (29.41) | 189 (29.21) | 193 (28.13) |
| No response | 3 (0.27) | 7 (0.62) | 0 (0.00) | 3 (0.27) | 4 (0.36) | 0 (0.00) | | 2 (0.36) | 0 (0.00) | 0 (0.00) | 1 (0.23) | 3 (0.61) | | 5 (0.86) | 0 (0.00) | 3 (0.46) | 3 (0.44) |
| Smoking status, *n*(%) |  |  |  |  |  |  | |  |  |  |  |  | |  |  |  |  |
| Non-smoker | 564 (50.90) | 624 (54.98) | 502 (57.63) | 700 (63.06) | 753 (67.35)** | 104 (16.91) | | 90 (16.19) | 69 (16.75) | 85 (18.36) | 106 (24.54)* | 460 (93.31) | | 534 (92.23) | 433 (94.34) | 615 (95.05) | 647 (94.31) |
| Formal-smoker | 190 (17.15) | 145 (12.78) | 133 (15.27) | 151 (13.60) | 138 (12.34) | 183 (29.76) | | 136 (24.46) | 127 (30.83) | 142 (30.67) | 134 (31.02) | 7 (1.42) | | 9 (1.55) | 6 (1.31) | 9 (1.39) | 4 (0.58) |
| Current-smokeer | 347 (31.32) | 356 (31.37) | 230 (26.41) | 252 (22.70) | 216 (19.32) | 328 (53.33) | | 328 (58.99) | 216 (52.43) | 236 (50.97) | 191 (44.21) | 19 (3.85) | | 28 (4.84) | 14 (3.05) | 16 (2.47) | 25 (3.64) |
| No response | 7 (0.63) | 10 (0.88) | 6 (0.69) | 7 (0.63) | 11 (0.98) | 0 (0.00) | | 2 (0.36) | 0 (0.00) | 0 (0.00) | 1 (0.23) | 7 (1.42) | | 8 (1.38) | 6 (1.31) | 7 (1.08) | 10 (1.46) |
| Physical activity, *n*(%) |  |  |  |  |  |  | |  |  |  |  |  | |  |  |  |  |
| None | 902 (81.41) | 944 (83.17) | 716 (82.20) | 918 (82.70) | 908 (81.22) | 492 (80.00) | | 451 (81.12) | 327 (79.37) | 367 (79.27) | 338 (78.24) | 410 (83.16) | | 493 (85.15) | 389 (84.75) | 551 (85.16) | 570 (83.09) |
| light (<1 h/day) | 170 (15.34) | 160 (14.10) | 127 (14.58) | 162 (14.59) | 160 (14.31) | 104 (16.91) | | 87 (15.65) | 69 (16.75) | 82 (17.71) | 68 (15.74) | 66 (13.39) | | 73 (12.61) | 58 (12.64) | 80 (12.36) | 92 (13.41) |
| moerate (<2 h/day) | 19 (1.71) | 17 (1.50) | 20 (2.30) | 18 (1.62) | 30 (2.68) | 10 (1.63) | | 9 (1.62) | 12 (2.91) | 11 (2.38) | 13 (3.01) | 9 (1.83) | | 8 (1.38) | 8 (1.74) | 7 (1.08) | 17 (2.48) |
| heavy (≥2 h/day) | 11 (0.99) | 5 (0.44) | 4 (0.46) | 8 (0.72) | 15 (1.34) | 7 (1.14) | | 4 (0.72) | 2 (0.49) | 2 (0.43) | 10 (2.31) | 4 (0.81) | | 1 (0.17) | 2 (0.44) | 6 (0.93) | 5 (0.73) |
| No response | 6 (0.54) | 9 (0.79) | 4 (0.46) | 4 (0.36) | 5 (0.45) | 2 (0.33) | | 5 (0.90) | 2 (0.49) | 1 (0.22) | 3 (0.69) | 4 (0.81) | | 4 (0.69) | 2 (0.44) | 3 (0.46) | 2 (0.29) |
| Menopause status, *n*(*%*) |  |  |  |  |  |  | |  |  |  |  |  | |  |  |  |  |
| Pre-menopause | ─ | ─ | ─ | ─ | ─ | ─ | | ─ | ─ | ─ | ─ | 259 (52.54) | | 317 (54.75) | 257 (55.99) | 402 (62.13) | 414 (60.35)* |
| Post-menopause | ─ | ─ | ─ | ─ | ─ | ─ | | ─ | ─ | ─ | ─ | 234 (47.46) | | 262 (45.25) | 202 (44.01) | 245 (37.87) | 272 (39.65) |
| Energy intake (kcal/day) | 1651.06 ± 479.48 | 1808.21 ± 539.53 | 1898.21 ± 521.33 | 2069.65 ± 582.11 | 2326.60 ± 665.83** | 1746.41 ± 479.50 | | 1915.63 ± 536.97 | 1975.28 ± 516.20 | 2164.10 ± 579.48 | 2407.19 ± 591.36** | 1532.10 ± 452.54 | | 1705.06 ± 521.99 | 1829.03 ± 516.74 | 2002.06 ± 574.98 | 2275.85 ± 704.43** |
| ^1^Values are means ± SD or n (%). *P*-trends were assessed by modeling the median value of the quintiles (continuous) or with the use of the Mantel-Haenszel chi-square test for linear trends (categorical). **P*-trend < 0.05; ***P*-trend < 0.0001. *iRFSH,* improved Recommended Food Score for Hypertension*; KoGES,* Korean Genome and Epidemiology Study; *SD*, standard deviation. | | | | | | | | | | | | | | | | | |

| **Supplemental Table 2** The mean score of each recommended food groups in iRFSH of the KoGES Ansan–Ansung study**^1^** | | | | | | | | | | | | | | | | |
| --- | --- | --- | --- | --- | --- | --- | --- | --- | --- | --- | --- | --- | --- | --- | --- | --- |
|  | Total (*n*= 5,342) | | | | | Male (*n*= 2,478) | | | | | | Female (*n*= 2,864) | | | | |
|  | Quintile 1 | Quintile 2 | Quintile 3 | Quintile 4 | Quintile 5 | Quintile 1 | Quintile 2 | Quintile 3 | Quintile 4 | Quintile 5 | Quintile 1 | | Quintile 2 | Quintile 3 | Quintile 4 | Quintile 5 |
| n | 1108 | 1135 | 871 | 1110 | 1118 | 615 | 556 | 412 | 463 | 432 | 493 | | 579 | 459 | 647 | 686 |
| Score range | 13 – 23 | 24 – 27 | 28 – 30 | 31 – 35 | 36 – 59 | 13 – 23 | 24 – 27 | 28 – 30 | 31 – 35 | 36 – 53 | 15 – 23 | | 24 – 27 | 28 – 30 | 31 – 35 | 36 – 59 |
| Score out of 65 | 20.55 ± 2.12 | 25.57 ± 1.15 | 28.94 ± 0.82 | 32.82 ± 1.40 | 40.10 ± 3.71** | 20.40 ± 2.22 | 25.54 ± 1.15 | 28.95 ± 0.82 | 32.76 ± 1.38 | 39.78 ± 3.42** | 20.75 ± 1.98 | | 25.60 ± 1.14 | 28.93 ± 0.83 | 32.87 ± 1.42 | 40.30 ± 3.87** |
| Whole grains, score out of 1 | 0.40 ± 0.49 | 0.51 ± 0.50 | 0.57 ± 0.49 | 0.58 ± 0.49 | 0.69 ± 0.46** | 0.34 ± 0.48 | 0.42 ± 0.49 | 0.52 ± 0.50 | 0.52 ± 0.50 | 0.63 ± 0.48** | 0.46 ± 0.50 | | 0.60 ± 0.49 | 0.62 ± 0.49 | 0.62 ± 0.49 | 0.73 ± 0.45** |
| Nuts and legumes, score out of 4 | 0.81 ± 0.77 | 1.23 ± 0.78 | 1.44 ± 0.83 | 1.64 ± 0.86 | 1.94 ± 0.89** | 0.90 ± 0.79 | 1.31 ± 0.78 | 1.49 ± 0.87 | 1.80 ± 0.88 | 2.08 ± 0.91** | 0.70 ± 0.72 | | 1.16 ± 0.77 | 1.39 ± 0.79 | 1.53 ± 0.82 | 1.86 ± 0.87** |
| Vegetables, score out of 25 | 4.47 ± 2.22 | 7.65 ± 2.51 | 9.70 ± 2.60 | 11.84 ± 3.13 | 15.74 ± 3.44** | 4.75 ± 2.35 | 8.25 ± 2.54 | 10.35 ± 2.55 | 12.58 ± 3.11 | 16.48 ± 3.47** | 4.11 ± 1.98 | | 7.07 ± 2.34 | 9.10 ± 2.49 | 11.30 ± 3.03 | 15.28 ± 3.35** |
| Fruits/Fruit juices, score out of 11 | 0.64 ± 1.13 | 1.44 ± 1.78 | 2.21 ± 2.16 | 3.57 ± 2.80 | 5.77 ± 3.14** | 0.60 ± 1.08 | 1.29 ± 1.69 | 1.95 ± 2.10 | 3.13 ± 2.82 | 5.16 ± 3.30** | 0.69 ± 1.18 | | 1.57 ± 1.85 | 2.45 ± 2.18 | 3.89 ± 2.75 | 6.16 ± 2.97** |
| Milk, score out of 1 | 0.33 ± 0.47 | 0.44 ± 0.50 | 0.53 ± 0.50 | 0.55 ± 0.50 | 0.67 ± 0.47** | 0.33 ± 0.47 | 0.42 ± 0.49 | 0.49 ± 0.50 | 0.51 ± 0.50 | 0.66 ± 0.47** | 0.33 ± 0.47 | | 0.45 ± 0.50 | 0.56 ± 0.50 | 0.58 ± 0.49 | 0.68 ± 0.47** |
| Fish, score out of 7 | 0.52 ± 0.89 | 1.09 ± 1.18 | 1.53 ± 1.29 | 2.05 ± 1.52 | 2.86 ± 1.67** | 0.60 ± 0.97 | 1.28 ± 1.29 | 1.64 ± 1.39 | 2.14 ± 1.55 | 3.08 ± 1.81** | 0.43 ± 0.76 | | 0.91 ± 1.04 | 1.44 ± 1.19 | 1.98 ± 1.49 | 2.72 ± 1.56** |
| Sugar-sweetened beverage, score out of 3 | 1.65 ± 0.57 | 1.63 ± 0.58 | 1.58 ± 0.62 | 1.50 ± 0.65 | 1.45 ± 0.70** | 1.53 ± 0.63 | 1.52 ± 0.64 | 1.45 ± 0.68 | 1.36 ± 0.71 | 1.29 ± 0.76** | 1.81 ± 0.44 | | 1.74 ± 0.50 | 1.70 ± 0.53 | 1.60 ± 0.59 | 1.56 ± 0.63** |
| Red and processed meat, score out of 7 | 6.30 ± 1.17 | 6.18 ± 1.21 | 6.00 ± 1.29 | 5.78 ± 1.46 | 5.61 ± 1.52** | 6.13 ± 1.26 | 5.93 ± 1.36 | 5.87 ± 1.39 | 5.56 ± 1.57 | 5.32 ± 1.64** | 6.52 ± 1.00 | | 6.42 ± 0.97 | 6.12 ± 1.19 | 5.93 ± 1.35 | 5.79 ± 1.41** |
| Sodium-rich food, score out of 6 | 5.18 ± 1.03 | 5.15 ± 1.05 | 5.10 ± 1.06 | 5.06 ± 1.10 | 5.07 ± 1.14* | 5.02 ± 1.08 | 4.91 ± 1.14 | 4.95 ± 1.13 | 4.92 ± 1.16 | 4.85 ± 1.23* | 5.38 ± 0.92 | | 5.38 ± 0.89 | 5.23 ± 0.97 | 5.16 ± 1.05 | 5.21 ± 1.06* |
| ^1^Values are means ± SD or n (%). *P*-for-trends were assessed by modeling the median value of the quintiles. **P*-trend < 0.05; ***P*-trend < 0.0001. *iRFSH,* improved Recommended Food Score for Hypertension*; KoGES,* Korean Genome and Epidemiology Study; *SD*, standard deviation. | | | | | | | | | | | | | | | | |

| **Supplemental Table 3** The mean and standard deviations of SBP and DBP across quintiles of iRFSH of the KoGES Ansan–Ansung study**^1^** | | | | | | | | | | | | | | | | | |
| --- | --- | --- | --- | --- | --- | --- | --- | --- | --- | --- | --- | --- | --- | --- | --- | --- | --- |
|  | Total (*n*= 5,342) | | | | | | Male (*n*= 2,478) | | | | | Female (*n*= 2,864) | | | | | |
|  | Quintile 1 | Quintile 2 | Quintile 3 | Quintile 4 | Quintile 5 | Quintile 1 | | Quintile 2 | Quintile 3 | Quintile 4 | Quintile 5 | Quintile 1 | Quintile 2 | Quintile 3 | Quintile 4 | Quintile 5 |  |
| *n* | 1108 | 1135 | 871 | 1110 | 1118 | 615 | | 556 | 412 | 463 | 432 | 493 | 579 | 459 | 647 | 686 |  |
| SBP |  |  |  |  |  |  | |  |  |  |  |  |  |  |  |  |  |
| Model 1^2^ | 113.99 ± 0.62 | 113.05 ± 0.62 | 112.47 ± 0.68 | 112.16 ± 0.61 | 111.43 ± 0.63** | 114.69 ± 0.82 | | 115.22 ± 0.72 | 113.62 ± 0.75 | 113.16 ± 0.67 | 113.18 ± 0.66* | 113.12 ± 0.69 | 112.94 ± 0.70 | 112.44 ± 0.63 | 111.76 ± 0.63 | 111.35 ± 0.61** |  |
| Model 2^3^ | 114.49 ± 0.64 | 114.60 ± 0.62 | 113.03 ± 0.66 | 112.72 ± 0.60 | 111.99 ± 0.61** | 115.14 ± 0.80 | | 115.31 ± 0.73 | 114.07 ± 0.75 | 113.64 ± 0.67 | 113.01 ± 0.68* | 113.67 ± 0.67 | 113.53 ± 0.71 | 113.10 ± 0.62 | 112.35 ± 0.60 | 111.92 ± 0.63** |  |
| DBP |  |  |  |  |  |  | |  |  |  |  |  |  |  |  |  |  |
| Model 1^2^ | 75.37 ± 0.64 | 74.81 ± 0.60 | 74.89 ± 0.62 | 74.54 ± 0.69 | 73.31 ± 0.67** | 77.44 ± 0.67 | | 77.21 ± 0.68 | 76.23 ± 0.64 | 76.72 ± 0.63 | 76.21 ± 0.70** | 74.06 ± 0.66 | 73.49 ± 0.65 | 73.7 ± 0.60 | 73.02 ± 0.62 | 73.13 0.61* |  |
| Model 2^3^ | 75.71 ± 0.63 | 75.60 ± 0.61 | 75.22 ± 0.57 | 74.30 ± 0.72 | 73.74 ± 0.68** | 77.73 ± 0.67 | | 76.57 ± 0.69 | 76.58 ± 0.67 | 75.07 ± 0.61 | 75.58 ± 0.69** | 74.44 ± 0.67 | 73.93 ± 0.63 | 73.14 ± 0.61 | 72.47 ± 0.63 | 72.57 ± 0.66** |  |
| ^1^Values are presented as means ± SD. *P*-trends were were assessed using general linear models for continuous variables after adjustment. ^2^Model 1: Unadjusted.  ^3^Model 2: adjusted by age, sex, BMI (underweight; normal; pre-obese; or obese class), education level(elementary school or less; middle school; high school; or college or above), household income (low; or high), drinking status (non-drinker; or current drinker), smoking status (non-smoker; former or current smoker), regular exercise (light activities; moderate-intensity activities; or heavy-intensity activities), energy intake (kcal/day), and menopause status (pre-menopause; or post-menopause for female only). **P*-trend < 0.05; ***P*-trend < 0.0001. *iRFSH*, improved Recommended Food Score for Hypertension; *KoGES*, Korean Genome and Epidemiology Study; *SD*, standard deviation; *SBP*, systolic blood pressure; *DBP*, diastolic blood pressure. | | | | | | | | | | | | | | | | | |

| **Supplemental Table 4** HRs and 95% CIs for hypertension according to the quintiles of iRFSH in the KoGES Ansan–Ansung study**^1^** | | | | | | | | | | | | | | | | | | | | | | | | | | | | | | | | | | | | | | | | | |
| --- | --- | --- | --- | --- | --- | --- | --- | --- | --- | --- | --- | --- | --- | --- | --- | --- | --- | --- | --- | --- | --- | --- | --- | --- | --- | --- | --- | --- | --- | --- | --- | --- | --- | --- | --- | --- | --- | --- | --- | --- | --- |
|  | |  | | | | | | | Total (*n*= 5,342) | | | | | | |  | | | | | | | Male (*n*= 2,478) | | | | | | |  | | | | | | | Female (*n*= 2,864) | | | | |
|  | | Continuous | | Quintile 1 | | Quintile 2 | | Quintile 3 | | | Quintile 4 | | Quintile 5 | | Continuous | | | Quintile 1 | | Quintile 2 | | Quintile 3 | | | Quintile 4 | | Quintile 5 | | Continuous | | | Quintile 1 | | Quintile 2 | | Quintile 3 | | | Quintile 4 | | Quintile 5 |
| *n* | 5342 | | 1108 | | 1135 | | 871 | | | 1110 | | 1118 | | 2478 | | | 615 | | 556 | | 412 | | | 463 | | 432 | | 2864 | | | 493 | | 579 | | 459 | | | 647 | | 686 | |
| Person-years, follow-up | 57896.6 | | 11958.3 | | 12232.8 | | 9548.3 | | | 12103.8 | | 12053.4 | | 26078.2 | | | 6477.1 | | 5872.2 | | 4285.5 | | | 4864 | | 4579.4 | | 31818.4 | | | 5481.2 | | 6360.6 | | 5262.8 | | | 7239.8 | | 7474 | |
| Model 1^2^ | 0.99  (0.88, 1.02) | | 1  (Reference) | | 0.99  (0.90, 1.08) | | 0.98  (0.89, 1.08) | | | 0.87  (0.79, 1.08) | | 0.84  (0.75, 1.10) | | 1.00  (0.95, 1.17) | | | 1  (Reference) | | 1.04  (0.92, 1.18) | | 1.06  (0.93, 1.22) | | | 1.10  (0.97, 1.25) | | 0.98  (0.86, 1.12) | | 1.01  (0.89, 1.09) | | | 1  (Reference) | | 0.98  (0.85, 1.12) | | 0.96  (0.83, 1.11) | | | 0.88  (0.77, 1.05) | | 0.85  (0.72, 1.02) | |
| Model 2^3^ | 0.98  (0.87, 1.03) | | 1  (Reference) | | 0.99  (0.89, 1.10) | | 0.98  (0.87, 1.09) | | | 0.92  (0.83, 1.03) | | 0.83  (0.74, 0.93)* | | 1.03  (0.98, 1.08) | | | 1  (Reference) | | 0.98  (0.85, 1.13) | | 0.96  (0.83, 1.13) | | | 1.05  (0.91, 1.21) | | 0.95  (0.82, 1.10) | | 0.98  (0.91, 0.99) | | | 1  (Reference) | | 1.00  (0.84, 1.18) | | 1.00  (0.83, 1.19) | | | 0.82  (0.69, 0.98) | | 0.73  (0.61, 0.88)* | |
| Model 3^4^ | 0.94  (0.90, 0.97) | | 1  (Reference) | | 1.00  (0.90, 1.11) | | 1.00  (0.89, 1.12) | | | 0.95  (0.84, 1.06) | | 0.79  (0.72, 0.87)** | | 0.99  (0.89, 1.07) | | | 1  (Reference) | | 1.00  (0.87, 1.15) | | 0.96  (0.82, 1.13) | | | 1.05  (0.90, 1.22) | | 0.95  (0.80, 1.11) | | 0.96  (0.86, 0.99) | | | 1  (Reference) | | 0.99  (0.82, 1.13) | | 1.04  (0.87, 1.26) | | | 0.84  (0.70, 0.97) | | 0.71  (0.62, 0.83)** | |
| ^1^Values are presented as HRs and 95% CIs. Only results for quintiles 1, 3, and 5 are shown; see Supplemental Table 4 for results across all quintiles. For calculating for *P*-trends, the RFSH was used in its continuous form. ^2^Model 1: Unadjusted.  ^3^Model 2: adjusted by age, sex, and menopause status (pre-menopause; or post-menopause for female only).  ^4^Model 3: adjusted by age, sex, BMI (underweight; normal; pre-obese; or obese class), education level (elementary school or less; middle school; high school; or college or above), household income (low; or high), drinking status (non-drinker; or current drinker), smoking status (non-smoker; former or current smoker), regular exercise (light activities; moderate-intensity activities; or heavy-intensity activities), energy intake (kcal/day), and menopause status (pre-menopause; or post-menopause for female only). **P*-trend < 0.05; ***P*-trend < 0.0001. *iRFSH*, improved Recommended Food Score for Hypertension; *KoGES*, Korean Genome and Epidemiology Study; *HR*, hazard ratio; *CI,* confidence interval. | | | | | | | | | | | | | | | | | | | | | | | | | | | | | | | | | | | | | | | | | |
